# The Role of ctDNA and Liquid Biopsy in the Diagnosis and Monitoring of Head and Neck Cancer: Towards Precision Medicine

**DOI:** 10.3390/cancers16183129

**Published:** 2024-09-11

**Authors:** Sami I. Nassar, Amber Suk, Shaun A. Nguyen, Dauren Adilbay, John Pang, Cherie-Ann O. Nathan

**Affiliations:** 1 Department of Otolaryngology—Head and Neck Surgery, Medical University of South Carolina, Charleston, SC 29425, USA; nassar@musc.edu (S.I.N.); nguyensh@musc.edu (S.A.N.); adilbay@musc.edu (D.A.); 2 Department of Otolaryngology—Head and Neck Surgery, Louisiana State University Health Sciences Center, Shreveport, LA 71103, USA; amber.suk@lsuhs.edu (A.S.); john.pang@lsuhs.edu (J.P.)

**Keywords:** head and neck cancer, liquid biopsy, ctDNA, squamous cell carcinoma, oropharyngeal carcinoma, nasopharyngeal carcinoma, genetic mutations, genomic sequencing, cancer biomarkers

## Abstract

**Simple Summary:**

Liquid biopsy’s use in the field of head and neck cancer has garnered interest due to providing an efficient and insightful alternative or complement to the present standards for the diagnosis and monitoring of patients. Certain biomarkers have been associated with specific cancer diagnoses, responses to treatment, and risk of recurrence, including circulating tumor DNA. Analysis of circulating tumor DNA and viral DNA via liquid biopsy has linked certain mutations and methylation patterns to various patient outcomes across several types of head and neck cancer. The present paper has reviewed the recent literature examining liquid biopsy’s clinical utility in head and neck oncology and has discussed the clinical implications of aspects of circulating tumor DNA, among other biomarkers.

**Abstract:**

Recent data have shown a continued rise in the worldwide annual incidence and mortality rates of head and neck cancers. The present standard for diagnosis and monitoring for disease recurrence or progression involves clinical examination, imaging, and invasive biopsy techniques of lesions suspected of being malignant. In addition to limitations relating to cost, time, and patient discomfort, these methodologies have inherent inaccuracies for detecting recurrence. In view of these limitations, the analysis of patient bodily fluid samples via liquid biopsy proposes a cost-effective and convenient alternative, which provides insight on the biogenetic and biomolecular underpinnings of oncologic disease processes. The monitoring of biomarkers for head and neck cancer via liquid biopsy, including circulating tumor DNA, circulating tumor cells, and circulating cell-free RNA, has shown clinical utility in the screening, diagnosis, prognostication, and monitoring of patients with various forms of head and neck cancer. The present review will provide an update on the current literature examining the use of liquid biopsy in head and neck cancer care and the clinical applicability of potential biomarkers, with a focus on viral and non-viral circulating tumor DNA. Possible future avenues for research to address specific shortcomings of liquid biopsy will be discussed.

## 1. Introduction

Head and neck cancer (HNC) refers to a pathologically varied group of malignant neoplasms of the upper aerodigestive tract and other structures of the head and neck regions [1]. Anatomic sites affected by HNC include the oral cavity, salivary glands, nasopharynx, oropharynx, nasal cavity, paranasal sinuses, larynx, skin of the head and neck, and, depending on the clinical definition, the thyroid gland [2,3]. The global incidence of HNC is predicted to continue to rise over the coming years, with trends indicating that human papilloma virus-related (HPV) oropharyngeal carcinomas will comprise the majority of HNCs over the next two decades [3]. From 2020 to 2022, cancers of the lip/oral cavity, larynx, nasopharynx, oropharynx, hypopharynx, and salivary glands rose from the 7th to the 6th most commonly diagnosed cancers and remained the 6th most common cancers to cause mortality worldwide. There are an additional 14,525 new HNC cases and 14,876 deaths per year associated with HNC being recorded in 2022 compared to malignancies of other anatomical sites [4,5].

The presence of nucleic acids in blood plasma was first reported by Mandel and Metais in 1948 [6]. Cell-free DNA (cfDNA) refers to genetic material organized in the form of DNA fragments of various sizes and from various origins found in bodily fluids independent of cells [7]. cfDNA can circulate free and unbound in the plasma, contained within a vesicle, or as part of macromolecular complexes composed of DNA bound to proteins or lipids [7]. DNA is released from cells into the plasma via several mechanisms, including apoptosis, necrosis, and NETosis, among others [8,9]. Given the role of cell damage and death in increasing the concentration of cfDNA in the serum, cfDNA has been recognized as a marker of inflammation and disease [9]. Accordingly, cfDNA has been found to be elevated in several pathological states, including certain kidney diseases, cardiovascular diseases, autoimmune conditions, and various cancers [10,11,12,13].

Circulating tumor DNA (ctDNA) was first explored by Leon et al. in 1977, and they found that participants with a variety of cancers had higher mean levels of cfDNA compared to healthy controls [13]. ctDNA refers to cfDNA derived from tumor cells [14]. ctDNA sequences are generally more fractionated and of shorter length than cfDNA fragments [14]. ctDNA is released from tumor cells into the bodily fluids either by cell death via apoptosis or necrosis or via active secretion [14]. In cancers of the head and neck, as well as cancers of other anatomical regions, the predominant pathways of ctDNA release into bodily fluids are via apoptosis and necrosis caused by rapid cell proliferation and tumor growth leading to tumor tissue hypoxia [15]. Hypoxia results from tumor growth outpacing the rate of neovascularization [15]. Certain treatment modalities, including surgery, radiotherapy, and chemotherapy, trigger cell death by causing tissue damage [16]. As the tumor cells die, impairment of phagocytosis by immune cells in the tumor tissue leads to accumulation of genetic material in the surrounding environment and subsequent release into nearby tissues [17]. As a result, the anatomic location of the tumor affects the concentration of ctDNA in different bodily fluids [17].

Quantitative and qualitative changes in the characteristics of ctDNA can provide essential information on a patient’s disease, thus having important clinical value [18]. For example, specific genetic mutations have been identified in various forms of cancer and can help inform diagnosis (Table 1) [18]. Methylation patterns have exhibited value in the early detection of cancer, predicting response to treatment, and determining cell of origin and therefore, cancer stage [19,20]. The concentration of ctDNA can be indicative of tumor burden, tumor localization, and rate of tumor growth [21].

Liquid biopsy allows for the analysis of ctDNA and other biomarkers of cancer present in the bodily fluids, including the blood, urine, saliva, and pleural, peritoneal, and cerebrospinal fluids [46]. The concept of liquid biopsy as an oncological diagnostic modality was first introduced in 2010 as a method for detecting circulating tumor cells (CTCs), and its application was expanded to the examination of ctDNA and other biomarkers in 2011 [47,48,49]. In HNC care, the current standard methods for diagnosis include endoscopy, imaging, and biopsy of a tumor sample by techniques such as excision or fine needle aspiration (FNA), while monitoring for recurrence is generally restricted to imaging and clinical examination (Table 2) [50,51,52]. The use of liquid biopsy has been successful in the diagnosis and monitoring of other solid tumors, and past studies on the use of liquid biopsy for the detection and monitoring of HNCs have shown potential for its clinical adoption as a less-invasive and less-expensive alternative to the current diagnostic standards [50,52]. The present literature review aims to review the literature on the implementation of liquid biopsy in HNC care, with a specific focus on ctDNA, and discuss its current and future use in relation to various histological types of head and neck malignancies.

## 2. Identification of Studies Included in the Present Review

PubMed (U.S. National Library of Medicine, National Institutes of Health), Scopus (Elsevier), Google Scholar, and Cochrane databases were reviewed from inception through 23 June 2024 to identify English-language articles discussing the use of liquid biopsy and ctDNA in the diagnosis and monitoring of various HNCs. ClinicalTrials.gov (U.S. National Library of Medicine, National Institutes of Health) was reviewed from inception through 3 September 2024, to identify clinical trials investigating liquid biopsy use in HNC care. Keywords used in the search strategies include combinations of “Head and Neck Cancer”, “Liquid Biopsy”, and “ctDNA”, as well as terms relating to anatomical and histological subtypes of HNCs, such as “Oropharyngeal”, “HPV”, and “Squamous Cell Carcinoma”. Titles and abstracts of the resulting articles were screened by the authors for relevance to the present review’s sections. Articles found to be relevant had their full texts reviewed. Findings and conclusions that were found to be pertinent to the present review were included.

Articles included in the present paper discuss the history of liquid biopsy’s development as a diagnostic and monitoring modality in the field of oncology, the various biomarkers that can be detected by liquid biopsy, and how these biomarkers can inform the clinical care of patients with various types of head and neck malignancies. Included articles were not limited to a specific type of research methodology. Articles that did not examine liquid biopsy in manners relevant to the topics discussed in the present literature review were excluded.

## 3. Liquid Biopsy

Initially, liquid biopsy assays focused on the analysis of CTCs but have since expanded to include ctDNA and circulating cell-free RNA (cfRNA) [49]. In oncology care, liquid biopsies are a minimally invasive method that can guide therapy, assess prognosis and tumor burden, and detect cancer by identifying and analyzing these molecules in a patient’s bodily fluid, most often blood [55]. While blood is the most common sample for liquid biopsy, saliva, cerebrospinal fluid, ascitic fluid, pleural fluid, and urine can be sampled as well.

**Figure 1:**
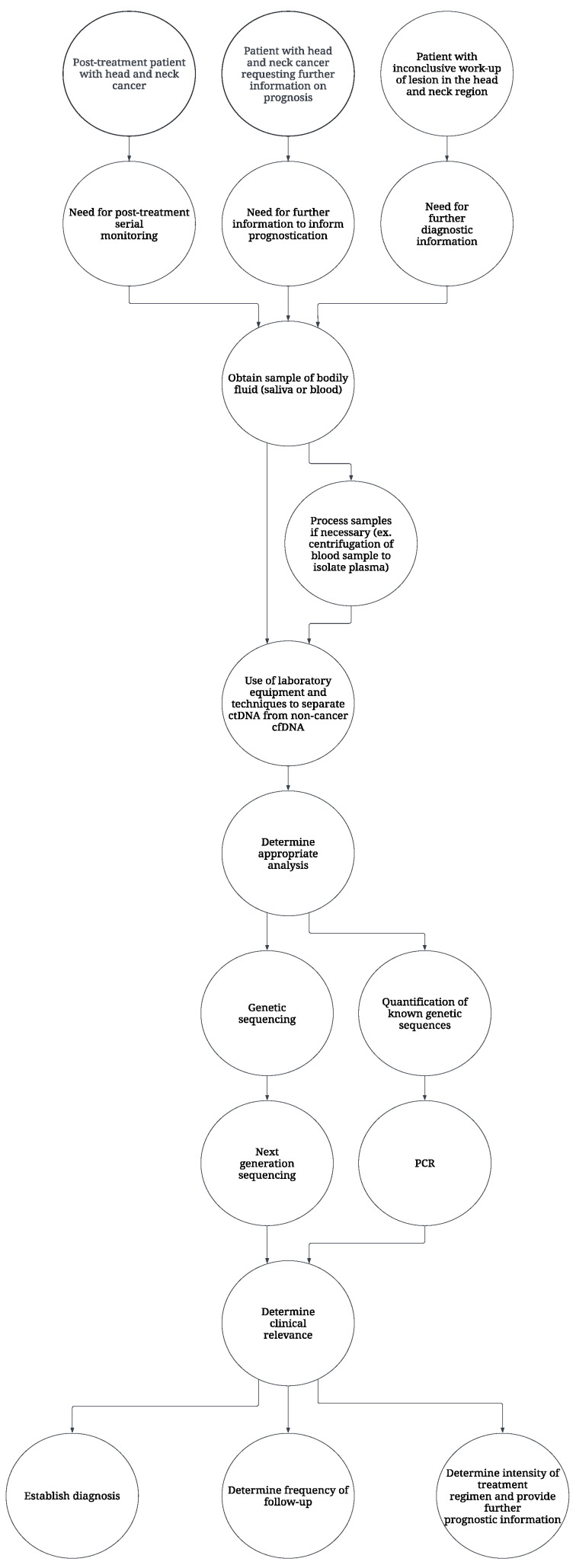
Use of liquid biopsy in HNC care.

The diagnostic workup for a patient with suspected HNC focuses on history, physical exam, and imaging, with tissue biopsies or cytology being used to establish a definitive diagnosis [56,57]. With the rapidly expanding field of genetic sequencing and tumor molecular profiling, liquid biopsies are being explored for their potential to improve outcomes among HNC patients [56,57,58]. In comparison to tissue biopsy or cytology, which are presently the gold standards for diagnosing head and neck squamous cell carcinomas (HNSCC), liquid biopsies are minimally invasive and may allow for earlier detection, prognosis, and monitoring of treatment response [55,56,57]. While blood, serum, or plasma are the most common samples used in liquid biopsy assays for HNC, technological advancements have led to the development of saliva-based assays [56,57]. A flowchart demonstrating the potential use of liquid biopsy techniques in clinical practice is shown in Figure 1.

There are a wide variety of biomarker assays being studied, including assays that detect and quantify CTCs, ctDNA, cfDNA, exosomes (EXOs), and tumor metabolites. Due to their rarity, CTCs can be difficult to isolate, but new 2-step isolation and purification techniques have been developed [56]. Other assays focus on non-cellular cancer components, like ctDNA or cfDNA, which are released by cancer cells or by macrophages that phagocytose them [56]. Assays must account for the varying levels of cfDNA, which depend on and are indicative of tumor burden, stage, and treatment [56]. EXOs are extracellular vesicles released by cells that measure between 30 and 150 nm and can be found in most bodily fluids [56]. The proteins, miRNA, mRNA, and DNA contained within an EXO’s lipid bilayer structure contribute to a tumor’s microenvironment and immune response [56]. EXO assays may have a higher yield than other assays; however, they can be more time-consuming to carry out and have a higher risk of contamination [56]. Finally, other tumor metabolites, like stearyl alcohol, sucrose, and plasma lysophosphatidylcholines, are being explored for their potential use as clinically relevant cancer biomarkers [56].

CTCs, while imperfect in certain respects compared to other biomarkers targetable by liquid biopsy, have been studied for their ability to prognosticate disease-free survival and overall survival in HNC patients and may have improved accuracy when used together with ctDNA [56,59]. Previously noted limitations associated with CTC use in HNC liquid biopsy assays compared to ctDNA and EXOs include very low concentrations of CTCs in bodily fluids, restriction of CTC detection to blood samples and absence of CTCs in other bodily fluids, and lower sensitivity values of available assays [56]. Detection rates of CTCs in patients with HPV-negative HNSCC and Epstein–Barr Virus-positive (EBV) nasopharyngeal carcinoma (NPC) were found to be highly variable and dependent on the assay used [59]. Conversely, the analysis of CTCs provides the opportunity to compare gene profiles of primary site tumor cells to those of CTCs and identify immune checkpoint markers expressed by CTCs that can inform immunotherapeutic interventions [56].

In addition to its use as a diagnostic and staging biomarker, ctDNA also has value in the monitoring of patients after treatment for the detection of disease recurrence, including when the patient is still asymptomatic [56]. Rutkowski et al. found that surveillance using a combination of ctDNA levels and PET-CT imaging may improve detection of recurrence of HPV-positive oropharyngeal cancer [60]. Furthermore, Lele et al. found that ctDNA detection in patient blood samples was significantly associated with post-treatment HNSCC recurrence, whereas identification of lesions with increased FDG uptake on PET scan was not, suggesting that liquid biopsy may be a superior modality for surveillance in certain cases [61].

**Figure 2:**
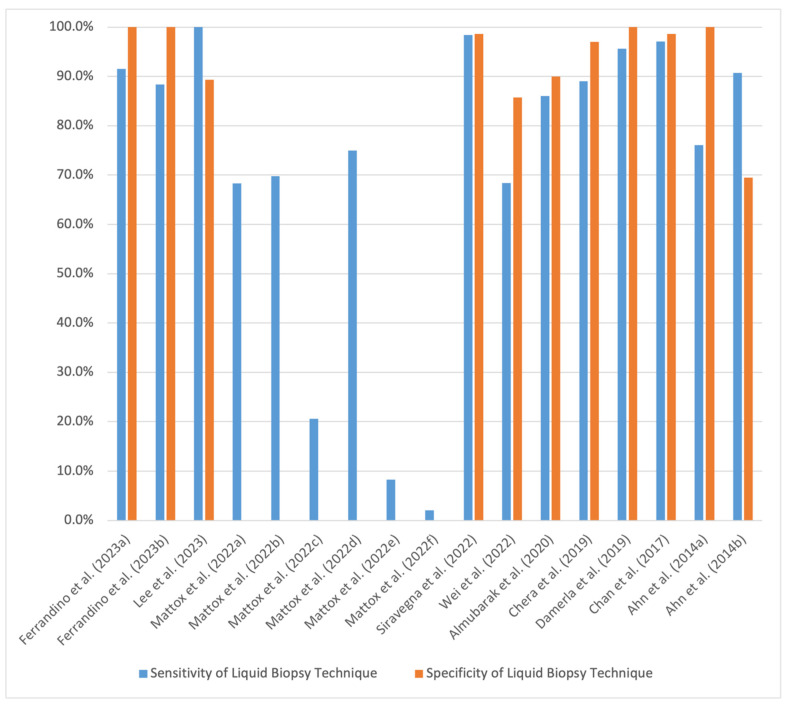
Reported sensitivity and specificity values of ctDNA in HNC care [41,42,44,63,64,65,66,67,68,69].

In comparison to traditional tissue biopsy, which remains the gold standard for diagnosis, liquid biopsy is less invasive and can allow for earlier diagnosis, more convenient monitoring post-treatment, and earlier detection of recurrence. In early stages of disease, when malignancies may not have yet formed a discrete recurrence, tissue biopsy is not feasible. Moreover, pulmonary nodules up to one centimeter in size are often too small for biopsy. While liquid biopsy can facilitate earlier detection in these stages, the concentration of biomarkers, including ctDNA, is low in early stages of disease, and assays are thus more susceptible to false positive results [62]. In combination with tissue biopsy, ctDNA and CTC assays may provide a more comprehensive understanding of a tumor’s biogenetic profile, thus further informing guide treatment plans [55]. For patients with HPV-associated oropharyngeal squamous cell carcinoma (SCC) that present with cystic or necrotic nodes, findings show that, while FNA only has a 70–80% success rate in identifying malignancy, ctDNA had a pooled sensitivity of 81% and specificity of 98% [63]. Specificity and sensitivity values of ctDNA liquid biopsy assays reported in the literature are showcased in Figure 2, and the characteristics of the respective studies are presented in Table 3.

For patients receiving immune checkpoint inhibitor treatment, imaging studies may show pseudo-progression of the tumor due to the increased inflammation. Studies examining various types of cancer have shown that ctDNA and cfDNA levels can be monitored to assess patient treatment response to immune checkpoint therapy, often providing greater insight than imaging studies [55]. For patients with HPV-positive HNSCC, measurement of circulating tumor HPV DNA (ctHPVDNA) identified treatment failure earlier than imaging (MRI, CT, and 18 F-FDG PET-CT) [70].

## 4. Head and Neck Squamous Cell Carcinoma

HNSCCs make up the majority of head and neck malignancies, with over 90% of HNCs being HNSCCs [22,71]. HNSCC can arise from the mucosal epithelium of the oral cavity, oropharynx, hypopharynx, larynx, and nasopharynx, with the main recognized risk factors for its development being tobacco use, alcohol use, and infection by high-risk HPV or EBV [22,71,72]. HPV infection has been identified as driving the increase in rates of oropharyngeal cancers in developed countries, with HPV-16 being the most common HPV type identified on biopsies of HPV-positive HNCs [3,73].

Early studies on the application of liquid biopsy to the treatment and monitoring of HNSCC found that CTCs were detectable in only a small number of cases, with increased stage and high tumor burden being associated with an increased number of CTCs in peripheral blood samples [74]. CTC counts were found to inform HNSCC tumor localization, potential dissemination secondary to treatment, and prognosis in terms of likelihood of disease-free survival, recurrence, and disease progression [74]. Studies on ctDNA found that increased ctDNA levels in samples also correlated with advanced stage and post-treatment recurrence rates; *TP53* was the most commonly identified mutated gene in HPV-negative HNSCC; ctDNA was detected in saliva samples of all patients with oral HNSCC; and combined plasma and saliva liquid biopsy was the most sensitive method for detecting ctDNA [74,75]. However, the paucity of data from early studies on liquid biopsy use in HNSCC diagnosis and monitoring, resulting from small sample sizes and large variations in the methodologies implemented and patient populations examined, limited the conclusions that could be drawn [74,75].

More recently, a systematic review by Huang et al. examining studies published between 2012 and 2023 corroborated earlier findings that *TP53* was the most commonly mutated gene in HNSCC [22]. *TP53* was also found to have the highest rate of concordant variants between tumor DNA (tDNA) and ctDNA at 6.25%, exhibiting the low degree of concordance in mutated genes detected in ctDNA and DNA of samples taken directly from the primary tumor site [22]. The highest degrees of concordance were detected in HPV-negative and stage IV HNSCCs [22]. The low concordance rate may be due to the high degree of intra-tumoral genomic heterogeneity between and within core and marginal sites of HNSCC tumors, with Payne et al. reporting that 96.5% of mutated genomic variants were found exclusively in a single site [76]. The examination of ctDNA via liquid biopsy in this same sample of patients was able to detect over 79% of high-frequency genomic variations, as well as most tumor site-specific mutations that could be missed by single-site tumoral biopsy, showcasing the potential of ctDNA liquid biopsy in both the diagnosis and tailoring of treatment of HNSCC according to genomic patterns identified [76]. Additionally, serial liquid biopsy testing of patients showed how frequencies of genomic variants and levels of intra-tumoral heterogeneity changed leading up to and at the time of HNSCC recurrence [76].

HPV-positive and HPV-negative HNSCCs differ in terms of commonly identified oncogenic mutations, which serve as identifiable targets for liquid biopsy. Data from The Cancer Genome Atlas (TCGA) has indicated that *PIK3CA* was the most commonly mutated oncogene in HPV-positive HNSCC, whereas genomic alterations in HPV-negative HNSCC were mostly limited to tumor suppressor genes, including *TP53*, *NOTCH1*, and *FAT1* mutations and *CDKN2A* inactivation [58,77,78,79,80]. While a previous study found that high *PIK3CA* copy number gain in HNSCC tumor samples was significantly associated with lower disease-specific survival and larger tumor volume, no studies have been found to date examining the prognostic value of *PIK3CA* mutations in the ctDNA of HNSCC patients [81]. The detection of *TP53* mutations in ctDNA has been associated with the presence of disease at last visit, regional metastasis, and decreased progression-free and overall survival, potentially making *TP53* a valuable biomarker for prognostication [23,24]. While *FAT1* mutations have been associated with better overall survival in HPV-negative HNSCC patients, their relationship to prognosis has not been examined in the exclusive context of ctDNA [82]. Lastly, *CDKN2A* and *NOTCH1* ctDNA mutations were not found to have any prognostic value [23]. Further research exploring the role of specific mutations present in the ctDNA of HNSCC and other HNC patients and their relation to prognosis is warranted.

While *PIK3CA* mutations are more frequent in HPV-positive HNSCCs compared to HPV-negative HNSCC, *PIK3CA* is also commonly mutated in HPV-negative HNSCC [83]. Another target for the detection of HPV-positive HNSCC via liquid biopsy is ctHPVDNA, which results from the integration of HPV DNA into the genome of the host–cell [65,84]. The use of digital droplet PCR (ddPCR) to detect HPV-associated E7 exhibits greater efficiency and accuracy in diagnosis compared to tissue biopsy while also increasing the ability to discriminate HNSCC according to HPV status [65]. Ferrandino et al. found that the use of liquid biopsy to test for tumor tissue-modified viral-HPV DNA in peripheral blood samples for diagnosis and testing for recurrence of high-risk HPV-positive oropharyngeal SCC had a sensitivity of over 88% and a specificity of 100% [63]. Saliva samples from HPV-positive HNSCC patients have also shown increased ctHPVDNA levels and have high concordance rates with peripheral blood samples [85]. In addition to saliva and blood samples, urine is another bodily fluid in which ctDNA can be detected. While the use of urine samples has previously been limited to detection of cancers of the urinary tract, transrenal ctDNA, which refers to ctDNA that passes from the bloodstream to the urine via filtration by the kidneys, has been shown to have diagnostic value in HNSCC [86]. A recent study by Bhambhani et al. found that ultrashort fragments comprising less than 50 base pairs, which are likely to be overlooked by traditional ctDNA assays, of HPV-16 transrenal ctDNA were detectable in patient urine samples by a newly developed ctDNA assay [86]. Testing for ctHPVDNA via liquid biopsy has been shown to have potential prognostic value, with ctHPVDNA levels exhibiting correlations with tumor burden, disease progression and metastasis, treatment response, residual disease, recurrence, and survival [84,85]. Preliminary data have also shown that ctHPVDNA has potential in the diagnosis of HPV-positive sinonasal and nasopharyngeal squamous cell carcinomas (SCC), which differ genotypically from HPV-positive oropharyngeal SCCs in that HPV-16 is less predominant compared to other HPV types [87].

E6 and E7, the two major HPV-related oncoproteins, affect DNA methylation patterns, with hypermethylation of specific genetic sequences being reported in HPV-positive HNSCC [88]. Of note, methylation of genes *CALML5*, *DNAJC5G*, and *LY6D* in ctDNA was highly effective in differentiating between HPV-positive oropharyngeal SCC patients and healthy controls and is an identifiable target in ctDNA studies [25]. Specifically, hypermethylation of *EDNRB* in ctDNA was found to be significantly associated with HNSCC when comparing a pooled group of HPV-positive and HPV-negative HNSCC patients to healthy controls; however, hypermethylation of *EDNRB* was only detectable in a minority of HNSCC patients, limiting its value as a diagnostic biomarker [26]. When considering methylation patterns in ctDNA of salivary samples, Lim et al. found that genes *RASSF1α*, *CDKN2A*, *TIMP3*, and *PCQAP/MED15* had higher levels of methylation in HPV-negative HNSCC patients compared to healthy controls, while the same genes had lower levels of methylation in HPV-positive HNSCC patients compared to healthy controls [27]. Other findings on methylation patterns in HNSCC previously described in the literature include associations between levels of primary tumor and ctDNA gene methylation levels, hypomethylation of Alu elements in ctDNA samples of HNSCC patients, and hypermethylation of gene promoter sequences in ctDNA of HNSCC patients, including the promoter sequences of *CDKN2A*, *CDKN2B*, *DAPK1*, *MGMT*, *GSTP1*, *PRDM2*, *RASSF1*, *DLEC1*, *UCHL1*, *RARβ2*, *WIF1*, *DCC*, *MLH1*, and *CDH1* [28]. Further elucidation of ctDNA methylation patterns and their implications in the characterization of HNSCCs will further inform the development of future diagnosis and treatment modalities.

## 5. EBV+ Nasopharyngeal Carcinoma

Nasopharyngeal carcinoma (NPC) is a rare cancer that originates from the mucosal epithelium of the nasopharynx and most commonly arises from the fossa of Rosenmüller [89]. Rates vary by geographical region, with over 70 to 80% of new cases being diagnosed in East and Southeast Asia [89,90]. Geographical differences in incidence and prevalence of NPC may be due to various environmental, genetic, viral, and dietary factors, as well as the interplay between them [91,92]. Of note, in endemic regions, EBV infection is implicated as contributing to carcinogenesis in about 96% of NPC cases [92].

Traditionally, NPC has been diagnosed via nasal endoscopy and direct sampling and biopsy of the lesion, followed by imaging scans for staging [53]. An early study examining cell-free EBV DNA (cfEBVDNA) was published in 1999, which found that cfEBVDNA was detectable via real-time quantitative PCR in the peripheral blood plasma samples of 55 of 57 patients with histologically confirmed diagnoses of NPC compared to 3 of 43 healthy controls [93]. Detected cfEBVDNA levels were significantly higher in patients with stage III/IV NPC compared to those with stage I/II NPC, and 7 of 15 patients demonstrated decreased cfEBVDNA following the completion of radiotherapy [93].

The diagnostic and prognostic utility of plasma cfEBVDNA has been further highlighted by findings showing significant correlations between plasma cfEBVDNA concentrations and gross tumor volume of the primary lesion and metastatic lymph nodes [94]. Overall survival, disease-free survival, distant metastasis-free survival, and distant metastasis are all significantly associated with the detection of pretreatment plasma cfEBVDNA, while posttreatment plasma cfEBVDNA levels are significantly associated with distant metastasis and locoregional recurrence [94,95]. It should be noted that plasma cfEBVDNA was preferable to MRI in detecting distant metastasis, whereas MRI was preferable to cfEBVDNA in detecting locoregional occurrence, highlighting the complementary roles that liquid biopsy and MRI could play in post-treatment monitoring of NPC [95].

It has been demonstrated that testing plasma samples for cfEBVDNA carries potential utility in screening for early-stage NPC in Hong Kong, where NPC is endemic [68]. Chan et al. found that 34 of the 309 participants had persistently elevated levels of plasma cfEBVDNA were ultimately diagnosed with NPC. Screened patients were diagnosed with early-stage disease at a greater proportion than in a previous study; screening was associated with greater progression-free survival; and screening for NPC via plasma cfEBVDNA had a sensitivity and specificity greater than 97% [68]. To differentiate between individuals with and without NPC who tested positive for cfEBVDNA in the plasma, a follow-up study molecularly profiled cfEBVDNA and found that NPC patients had generally longer fragment lengths than their non-NPC counterparts [96]. While promising, it has been argued that screening for NPC via quantification of cfEBVDNA should be used in conjunction with other screening modalities: an estimated 130 patients with NPC would be missed annually in Hong Kong if plasma cfEBVDNA was implemented as the sole population screening tool [97].

Several DNA sequences have been identified as targets for hypermethylation in samples from patients with NPC. Higher rates of gene-silencing EBV-associated hypermethylation of CpG islands have been noted in the cfDNA promoter sequences of tumor suppressor genes *RASSF1*, *CDKN2A*, *CDKN2B*, *DLEC1*, *DAPK1*, *UCHL1*, *WIF1*, *RARβ2*, and *CDH1* of NPC patients compared to healthy controls [29,30,31,32]. ctDNA analysis of peripheral blood samples shows the highest levels of methylation in patients with metastatic NPC of the gene bodies and intragenic regions of chromosomes 1 and 2, with greater hypermethylation levels of the open sea region compared to other regions of the CpG islands, which differs from DNA hypermethylation patterns of NPC tumor tissue [33]. More specifically, genes *PLCB3*, *C18orf1*, *ZNF516*, *FGR*, *PLCB3*, *FGR*, *PRKCZ*, *KDM4B*, *HLX*, *MGRN1*, *UHRF1*, *SPI1*, *PLEC1*, *MPO*, *ADRBK1*, *COL11A2*, *MLLT1*, *FUT4*, *MBP*, and *FLNB* were hypermethylated, whereas genes *SMTN*, *KCNT1*, *APEH*, and *HLA-DRB5* were hypomethylated [33]. Quantitative PCR of NPC patient saliva samples was also able to reliably differentiate between patients with NPC and healthy controls on the basis of cfEBVDNA CpG island methylation levels, with decreases and increases in methylation levels relative to the cut-off value of the assay after therapy and after recurrence, respectively [98]. Given these findings, hypermethylation patterns of ctDNA carry potential for prognosis in addition to diagnosis.

Other biomarkers for NPC include CTCs, whose levels in the peripheral blood have been found to positively correlate with N stage and overall clinical stage and negatively correlate with survival in patients with stage III-IVA NPC [99]. Post-chemoradiotherapy, mesenchymal CTC detection in peripheral blood samples was positively correlated with N stage and negatively correlated with 3 year survival [99]. MicroRNAs, which are approximately 22 nucleotide RNA fragments, encoding the BART region of EBV DNA are tissue-specific and can be released into the bloodstream similarly to ctDNA, with suggestions that BART microRNA could be used for the detection of NPC due to being present in higher levels in the peripheral blood of NPC patients compared to healthy controls [53,100]. Lastly, there is recent evidence that tumor-educated platelet-long non-coding RNA regulators of reprogramming plasma levels are negatively associated with NPC and carry a similar diagnostic value to cfEBVDNA [101].

## 6. Other Types of Head and Neck Cancers

Salivary gland primary tumors are rare and most commonly affect the parotid gland, although they can also arise from the smaller salivary glands, in which case they are generally malignant [102]. Diagnosis is performed via FNA and subsequent histological analysis, yet diagnosis can be challenging due to the histological heterogeneity of salivary gland neoplasms and the difficulty to differentiate between benign and malignant tumor cells on cytology [102]. Consequentially, determination of the neoplasm’s histology is typically performed post-surgery via immunohistology [102].

Several potentially targetable biomarkers for the detection and monitoring of salivary gland cancers via liquid biopsy have been explored; however, there is a paucity of previous literature on the topic. Genomic profiling of ctDNA in a set of patients with various histological types of salivary gland carcinomas determined the most commonly altered genes to be *TP53*, *PIK3CA*, *ERBB2*, *ATM*, *EGFR*, and *HRAS*, while *BRAF* and *KRAS* mutations and *EGFR* amplification were identified as potentially targetable alterations that were newly detected on serial testing [34]. When examining ctDNA alterations by histological subtype of salivary gland carcinoma, it was found that *PI3KCA* mutations were common in adenoid cystic carcinoma and salivary duct carcinoma, *ERBB2* mutations were common in salivary gland adenocarcinoma, and *EGFR* mutations were common in salivary gland mucoepidermoid carcinoma [34]. Additionally, in patients with metastatic adenoid cystic carcinoma, copy number analysis detected chromosomal alterations in ctDNA that were consistent with genetic mutations from samples taken from the primary tumor site, and *CDK6* gene amplification in chromosome 7q was identified in peripheral blood samples as a potential biomarker for disease progression [35]. Other identified potential biomarkers include elevated plasma levels of IL-33 in benign and malignant salivary gland neoplasms and its receptor, sST2, among metastatic acini cell carcinomas and benign pleomorphic adenomas, elevated levels of IL-4 in the peripheral blood of patients with malignant salivary duct carcinomas, and elevated levels of CA 19-9 in salivary samples of patients with malignant parotid cancers [102]. Preliminary studies have also noted that increased levels of expression of the androgen receptor splicing variant ARv7 by CTCs in peripheral blood samples of a single patient with metastatic salivary gland carcinoma predicted treatment resistance to combined androgen blockade therapy, and that detection of CTCs in peripheral blood samples of patients with adenoid cystic carcinoma could indicate local recurrence or distant metastasis [103,104].

Sinonasal cancers are rare tumors of the head and neck that may present with nasal and neurological symptoms, and they carry a significant risk of metastasis and local recurrence [54]. Current recommendations state that sinonasal cancers should be diagnosed via endoscopy and tumor biopsy followed by imaging with CT, MRI, and 18 F-FDG PET/CT to evaluate localization, histology, and size of the tumor, invasion into nearby bony and soft tissue structures, and presence of metastasis [54]. As noted in past literature, data on the utility of liquid biopsy in the diagnosis and monitoring of sinonasal cancers is sparse [59]. Micro RNA has been identified as a potential indicator of disease progression in patients with sinonasal intestinal-type adenocarcinomas (ITAC), with levels of miR-34c levels in nasal washings being increased in patients with increased levels of tumor differentiation and decreased in patients with signs of tumor intracranial extension, orbital extension, and advanced tumor staging [105]. Additionally, Cabezas-Camarero et al. discussed a case of the use of liquid biopsy to detect *KRAS* mutations in exon 2 codon 12 of CTC DNA in the peripheral blood of a patient with recurrent and anti-EGFR therapy-resistant ITAC, which were concordant with mutations found in the solid tumor biopsies via BEAMing, a technique that was previously found effective in detecting colorectal cancer-related genetic mutations [36]. A case series by Freiberger et al. on three patients with immunotherapy-resistant sinonasal melanoma found that *NRAS* mutations arose in the ctDNA collected from plasma samples during or after finishing treatment [37]. Lastly, two of three patients with sinonasal cancer who had CTCs detected in their peripheral blood samples had locally advanced sinonasal differentiated carcinoma [106]. While these findings may suggest promise in the use of liquid biopsy in the care of patients with sinonasal cancers, larger scale studies need to be carried out before broader conclusions are made.

Thyroid cancer refers to various malignancies that originate from thyroid tissue follicular cells, or C-cells, and differ in regard to histology and clinical implications [38]. The current standard for the diagnosis of thyroid cancers is detection by ultrasound imaging followed by confirmation via fine needle aspiration cytology (FNAC); however, in addition to being invasive, FNAC is operator-dependent and results in indeterminate findings in 15–30% of cases, and is limited in differentiating between benign follicular adenomas and malignant follicular carcinomas [39]. Monitoring for recurrence of thyroid cancer post-total thyroidectomy is carried out by serial measurements of serum thyroglobulin levels, but this is limited by variations in results of different assays and the interference of serum thyroglobulin antibodies [39]. Liquid biopsy has the potential to address these limitations.

Genomic patterns of plasma ctDNA samples have been found to vary between patients with different types of thyroid cancer, with *TP53* being the most commonly mutated across histological subtypes [38]. Other altered genes included *BRAF*, *RAS*, *RET*, *ALK*, *NTRK*, *PIK3CA*, and *PTEN* [38,39,40]. Specifically, one study reported that the *BRAF^V600E^* mutation has been detected in the serum samples of some patients with papillary thyroid cancer but was not found to be associated with lymphatic invasion, lymph node metastasis, or extra-nodal extension [41]. Conversely, the prognostic utility of *BRAF^V600E^*-mutated ctDNA is supported by the findings of Almubarak et al., who found that *BRAF^V600E^*-mutated ctDNA levels were higher in patients with metastatic compared to non-metastatic papillary thyroid carcinoma (PTC) [42]. Additionally, ctDNA serum levels were found to have a higher sensitivity and specificity (86% and 90%, respectively) in diagnosing PTC than the standard thyroglobulin assay (78% and 65%, respectively) [42]. Hypermethylation of *RASSF1* and *SLC5A8* promoter regions was found to be higher in both tumor tissue samples and ctDNA of patients with PTC compared to those with benign thyroid nodules, and hypermethylation of the promoter regions of *SLC5A8* in ctDNA was significantly associated with stage of PTC [43]. Another study reported that concordance of *BRAF^V600E^*-mutated plasma ctDNA and primary lesion DNA collected via FNAC was noted in 73.08% of a cohort of participants composed of 22 patients with PTC and four comparators with benign thyroid nodules, and that five out of six patients with PTC that were positive for *BRAF^V600E^*-mutated ctDNA were found to be negative 24 h post-surgery [44]. Yet, this is not the case across thyroid cancer subtypes: A study on patients diagnosed with medullary thyroid carcinoma found that detection of mutually exclusive *RET* and *RAS* mutations in ctDNA did not significantly associate with patients being pre- or post-surgery [45]. In terms of anaplastic thyroid carcinoma, the most aggressive subtype of thyroid cancer, Sandulache et al. reported that the most commonly detected ctDNA mutations in a sample of 23 patients were *TP53* and *BRAF* (65% and 48%, respectively), and treatment-naive patients had higher concordance rates between ctDNA and tDNA [40].

Apart from ctDNA, other biomarkers that can be targeted by liquid biopsy include CTCs and various types of cfRNA. CTC detection and monitoring has been claimed to be effective in distinguishing between benign and malignant thyroid nodules, distinguishing between patients with differentiated thyroid cancer and healthy controls, determining initial tumor stage, prognosticating patients with metastatic disease, determining primary tumor size and presence of vascular invasion, and monitoring patient response to radioiodine therapy [39,107]. cfRNA had utility in diagnosis, identification of recurrence, staging, prognostication, and informing treatment in several types of thyroid cancer [39].

## 7. Challenges and Limitations

Although promising, the use and implementation of liquid biopsy in HNC care is not without challenges and limitations. Several technological advancements in liquid biopsy techniques have allowed for increased effectiveness and precision in its use as a diagnostic and monitoring modality. For example, next-generation sequencing (NGS) and ddPCR were found to have greater sensitivity than qualitative PCR in detecting ctHPVDNA in patient plasma samples, while NGS had greater sensitivity than ddPCR and qualitative PCR in detecting ctHPVDNA in oral rinses of patients with HPV-associated HNSCC [64]. Furthermore, NGS of ctDNA in blood samples of patients with various types of recurrent and metastatic HNCs identified new genetic mutations not detected on NGS of tDNA [108]. In addition to NGS of plasma having a higher sensitivity in the detection of genetic mutations than NGS of tumor tissue, it is also less invasive [108,109]. However, high costs and convoluted data analyses of technologies like ddPCR and NGS present a barrier for the widespread clinical adoption of liquid biopsy [110].

Other issues relating to costs include the need for adequate materials and infrastructure for the storage of patient samples, which could affect the reliability and accuracy of liquid biopsy if suboptimal [111]. For example, ddPCR requires certain reagents with high costs to function [58]. Additionally, carrying out higher-order multiplexing, in which ddPCR quantifies the amount of more than two targets within a single reaction, is complicated and would require further training of laboratory technicians responsible for performing these techniques [58,112]. Nevertheless, costs and convoluted data manipulation, among other factors limiting the accessibility of these technologies and their use in routine clinical care, have been identified, and efforts are being undertaken to address them [51,57,110]. For example, Nonaka and Wong have developed a diagnostic liquid biopsy alternative to traditional assays, known as EFIRM (electric field-induced release and measurement), which requires low volumes of bodily fluids and can reliably detect ctDNA and exosomal RNA [110]. Proposals have been made to require further computational training for scientific researchers to obtain the necessary skills for the management and analysis of complicated data originating from NGS, and this suggestion should also apply to personnel responsible for the analysis of results of assays used in clinical care [113]. In addition to training interventions, user-friendly software has been developed to facilitate the management of NGS data and overcome barriers associated with it, such as the expensive computational infrastructure required for NGS data analyses [114]. Future efforts to further increase the accessibility of these technologies will be necessary if liquid biopsy is to become widespread in clinical adoption.

Another barrier that has been identified is the necessity for more data from prospective, longitudinal studies on the clinical utility of liquid biopsy to ascertain the effectiveness of various assays in patient care [115]. Such studies could help refine the sensitivity and specificity values of these assays, which have been noted by some to be clinically suboptimal [56,58]. Furthermore, in specific relation to the diagnosis and monitoring of HPV-related HNSCC, improvement of current technologies is warranted to expand the currently limited set of HPV types that are able to be detected via liquid biopsy assays [116]. The lack of FDA-approved, HNC-related liquid biopsy biomarkers poses another challenge that prevents its adoption for clinical use [58].

Other considerations to note concern disease-, treatment-, and data collection-related limitations of liquid biopsy. Due to the tumor heterogeneity-related discordance often found between circulating biomarkers and those of the primary tumor tissue, liquid biopsy may be limited in its ability to comprehensively characterize malignancies from which the analyzed biomarkers originate [76,110]. Furthermore, recurrence at primary sites may be less detectable, especially in the setting of altered lymphatics. Additionally, variables including undergoing radiochemotherapy, gastrostomy tube placement, having an infection, and antibiotic use were associated with increased cfDNA levels in blood samples of HNC patients, which could be confounding variables in the use of liquid biopsy for the monitoring of treatment response and cancer recurrence [117]. The capability of liquid biopsies to distinguish between biomarkers originating from cancerous and non-cancerous tissues poses another hurdle [56]. Given that multiple factors contribute to levels of cfDNA detected in patient bodily fluids, the clinical context should be accounted for when considering the implications of liquid biopsy test results. Serial repetition of cfDNA concentration measurements would help discern between transient inflammatory states, such as a spike in detected ctDNA levels following recent radiotherapy, and cancer recurrence or persistence [9]. The delineation of cfDNA levels associated with certain inflammatory states and severity of inflammation, use of complementary testing and imaging in addition to liquid biopsy, establishing the baseline ctDNA concentration of patients prior to and following treatment for comparative use in monitoring, and identifying mutations or genetic sequences that are malignancy-specific and can be tested for and reliably detected can potentially mitigate these limitations [9,117,118].

There are other limitations intrinsic to the use of ctDNA and current liquid biopsy technologies. False positive and negative test results have been discussed, and this has clinical implications that can affect treatment plans and cause distress among patients [119,120,121]. To address variations in sensitivities of different assays, multigene panel analyses of ctDNA have been suggested [119]. Similar concerns regarding the sensitivity and specificity of using other biomarkers in liquid biopsy assays, such as CTCs, have been identified and are currently being researched [121]. Other challenges identified that are necessary to address prior to widespread clinical adoption of liquid biopsy technologies include the need to establish practical standards regarding the optimal biological samples to be used, methods of sample storage, and clinically relevant biomarkers to be tested for, which may differ according to cancer histopathology or anatomical location [119]. There is also the need to standardize preanalytical variables to control for confounding effects on assay results and perform studies comparing various liquid biopsy assays to identify which are most appropriate in specific clinical scenarios [122].

It should be noted that ctDNA in peripheral blood samples comprises a small fraction of the cfDNA present in circulation, with the majority of cfDNA originating from hematopoietic cells [120]. Thus, non-cancer cfDNA may dilute the amount of circulating genetic material directly relevant to oncological care [120]. This is further compounded by the possibility of low amounts of tumor shedding related to various factors, including tumor burden and location [123]. The cfDNA originating from these hematopoietic cells may gain somatic mutations known as clonal hematopoiesis of indeterminate potential (CHIP) mutations [120,123,124]. This would contribute to complicating the ability to differentiate between oncogenic mutations in ctDNA and spontaneous somatic mutations present in non-cancer cfDNA, thus increasing the risk for false positive results [120,123,124]. Suggestions to reduce the amount of potentially confounding nontumorous cfDNA in liquid biopsy analytes include maintaining samples at ambient temperatures, performing double plasma centrifugation and using special cfDNA blood collection tubes to separate different-size fragments of cfDNA from one another, performing laboratory testing soon after sample collection, sequencing normal leukocytes to identify CHIP mutations, and avoiding sample collection in periods of heightened cfDNA levels [124]. Furthermore, low levels of ctDNA are present in bodily fluids in the early stages of cancer, which may lead to false negative test results due to ctDNA needing to comprise at least ten percent of the cfDNA to provide accurate information on tumor characteristics [125]. While increasing the sensitivity of liquid biopsy assays by expanding upon the depth of sequencing may seem like a potential solution, false-positive rates would increase due to genetic sequences of oncogenic mutations being detected in cfDNA not originating from tumors [125].

Furthermore, the management algorithm for patients with early recurrences detected via liquid biopsy without clinical or radiographic evidence of disease has yet to be established. Haring et al. argue that liquid biopsy may be limited in its ability to characterize the burden or location of disease recurrence [126]. For example, there are no agreed-upon ctHPVDNA levels to differentiate between local, regional, and distant recurrences [127]. This limits the effectiveness of liquid biopsy in informing therapeutic interventions, which differ in appropriateness depending on location of recurrence [126]. In terms of surveillance schedules, positive liquid biopsy testing without clinicoradiological correlates can prompt more frequent testing and imaging to ensure earlier detection of clinical disease [128]. Clinical trials and other future investigations will quantify the utility of liquid biopsy in this regard.

The ethical and psychological implications of liquid biopsy’s technical limitations must also be noted. False liquid biopsy results stemming from the suboptimal reliability of certain assays may cause undue psychological distress to patients [129]. False positives can increase patient stress, anxiety, and fear of death while also subjecting patients to the psychological and physical effects of unnecessary treatment [129]. False negatives can lead to delays in necessary care and treatment, thus decreasing chances of positive patient outcomes and leading to further future deterioration in patient quality of life due to unchecked disease progression [129]. Additionally, the lack of clear clinical guidelines on how to proceed in patients with recurrences detected via liquid biopsy without clinical or radiographic evidence of disease, as was discussed above, can contribute to patient anxiety secondary to increased patient uncertainty [130]. On the other hand, liquid biopsy has the potential of decreasing patient discomfort and distress by offering a less invasive alternative to current diagnostic and monitoring approaches, decreasing financial burden and associated stress by early identification and treatment of disease, and reassuring patients by providing greater insight on their disease and prognosis [129,131].

## 8. Conclusions and Future Directions

Liquid biopsy carries the potential to revolutionize HNC care. It has shown promise in diagnosis, staging, prognosis, and treatment monitoring of various types of HNCs; however, more research is warranted to resolve contradictions presented by the results of different studies and elucidate liquid biopsy’s clinical applicability and utility. Recent clinical trials exploring the use of liquid biopsy in HNC care are listed in Table 4.

Future avenues for investigative studies include determining ways to simplify and increase accessibility and affordability of liquid biopsy assays, refining current technologies to ensure adequate clinical standards are met, and identifying other disease-specific biomarkers and elucidating the implications of their detection at different points in a patient’s disease course. In terms of accessibility, specific barriers have been identified that may limit underserved populations’ access to liquid biopsy. Barriers faced by patients from underserved backgrounds include providers being less likely to recommend new technologies to these patients, patient mistrust of the medical establishment and decreased willingness to be subject to emergent medical techniques, and poor health literacy impeding the understanding of the pros and cons of liquid biopsy [131]. In addition to recruiting samples representative of the HNC patient population at large for enrollment in clinical trials, complementing clinical trial findings with findings from studies examining the utility of liquid biopsy among larger and more generalizable study populations can provide valuable insight on the large-scale applicability and utility of liquid biopsy [131]. With the continued refinement and development of liquid biopsy assays, the establishment of clinical guidelines and standards, and the identification of clinically relevant biomarkers, liquid biopsy promises to increase the possibilities for individualized and personalized approaches to HNC patient care and oncology care at large.

## Figures and Tables

**Table 1:** Identified altered genes in ctDNA by type of malignancy.

Type of Malignancy	Identified Altered ctDNA Genes
Head and neck squamous cell carcinomas [22,23,24,25,26,27,28]	- *TP53* - *NOTCH1* - *CDKN2A* - *CALML5* - *DNAJC5G* - *LY6D* - *EDNRB* - *TIMP3* - *PCQAP/MED15* - *CDKN2B* - *DAPK1* - *MGMT* - *GSTP1* - *PRDM2* - *RASSF1* - *DLEC1* - *UCHL1* - *RARβ2* - *WIF1* - *DCC* - *MLH1* - *CDH1*
Nasopharyngeal carcinoma [29,30,31,32,33]	- *RASSF1* - *CDKN2A* - *CDKN2B* - *DLEC1* - *DAPK1* - *UCHL1* - *WIF1* - *RARβ2* - *CDH1* - *PLCB3* - *C18orf1* - *ZNF516* - *FGR* - *PLCB3* - *PRKCZ* - *KDM4B* - *HLX* - *MGRN1* - *UHRF1* - *SPI1* - *PLEC1* - *MPO* - *ADRBK1* - *COL11A2* - *MLLT1* - *FUT4* - *MBP* - *FLNB* - *SMTN* - *KCNT1* - *APEH* - *HLA-DRB5*
Salivary gland carcinomas [34,35]	- *TP53* - *PIK3CA* - *ERBB2* - *ATM* - *EGFR* - *HRAS* - *BRAF* - *KRAS* - *CDK6*
Sinonasal carcinomas [36,37]	- *KRAS* - *NRAS*
Thyroid carcinomas [38,39,40,41,42,43,44,45]	- *TP53* - *BRAF* - *RAS* - *RET* - *ALK* - *NTRK* - *PIK3CA* - *PTEN* - *RASSF1* - *SLC5A8*

**Table 2:** Current standards for the diagnosis of head and neck cancers.

Type of Malignancy	Diagnostic Standard
Head and neck squamous cell carcinomas [51]	- Imaging - Endoscopy - Primary tumor biopsy and histological analysis - FNA with ultrasound guidance in hard-to-access areas
Nasopharyngeal carcinoma [53]	- Primary tumor biopsy with assistance via nasal endoscopy - Imaging
Salivary gland carcinomas	- FNA and cytological analysis; if unclear, immunohistology of excised tumor tissue can establish diagnosis
Sinonasal carcinomas [54]	- Imaging with both CT and MRI - Endoscopy - Primary tumor biopsy - “Metabolic biopsy” via 18 F-FDG PET/CT
Thyroid carcinomas [39]	- FNA with ultrasound guidance

**Table 3:** Studies examining clinical applications of liquid biopsy assays in HNC care.

Study	Aim	Type of Cancer	Sample Size	Method and Sample Used	Sensitivity of Liquid Biopsy Technique	Specificity of Liquid Biopsy Technique	Method Compared to	Conclusions	Limitations
Ferrandino et al. (2023) [63]	Determining performance metrics of ctHPVDNA in the diagnosis of oropharyngeal SCC	Oropharyngeal SCC	163; 152 with HPV-positive SCC and 11 with HPV-negative SCC	TTMV-HPV DNA testing; plasma	91.5%	100%	Using tumor samples, p16 staining confirmed diagnosis in 98.7% of patients, HPV PCR in 75%, and in situ hybridization assays in 10.5%	Approximately 1 in 10 false negatives will result; ctHPVDNA assays should be used in conjunction with other tests	Ascertainment bias as patients were known to have HPV-positive oropharyngeal SCC
	Determining performance metrics of ctHPVDNA in the detection of recurrence of HPV-positive oropharyngeal SCC at 3 months post treatment completion	HPV-positive oropharyngeal SCC	290	TTMV-HPV DNA testing; plasma	88.4%	100%	Using tumor samples, p16 staining confirmed diagnosis in 95.5% of patients, HPV PCR in 84.8%, and in situ hybridization assays in 4.8%		Prospective study where most patients did not have pretreatment ctHPVDNA measurements available; conservative definition of false-negative recurrence at 3-month follow-up potentially lowered the calculated sensitivity of the assay
Lee et al. (2023) [41]	Detecting multifocality of papillary thyroid carcinoma (PTC)	PTC	37	PDA/ SiO_2_-coated bead cfDNA detection assay; serum	100%	89.3%	Free T4 had a sensitivity of 33.3% and specificity of 82.1%; TSH had a sensitivity of 66.7% and specificity of 60.7%; Tg had a sensitivity of 11.1% and specificity of 64.3%; TgAb had a sensitivity of 33.3% and specificity of 82.1%	PDA/SiO_2_-coated bead liquid biopsy assays effectively capture ctDNA that can permit analysis of multiple mutations associated with PTC	Small sample size, no control subjects
Mattox et al. (2022) [64]	Comparing sensitivity of NGS, ddPCR, and qPCR assays in the detection of ctHPVDNA in plasma and oral rinse samples	HPV-16-positive oropharyngeal SCC	66	NGS; plasma	68.3%	Not reported	Compared qPCR, ddPCR, and NGS sensitivity values for analysis of plasma and oral rinse samples	NGS and ddPCR have significantly higher sensitivity values for the detection of ctHPVDNA in plasma samples compared to qPCR, while NGS has a significantly higher sensitivity value for the detection of ctHPVDNA in oral rinse samples compared to ddPCR and qPCR. Levels of ctHPVDNA detected by NGS in plasma samples may reflect the clinical course of patients with HPV-positive oropharyngeal SCC	Small sample size, no control subjects
				ddPCR; plasma	69.8%				
				qPCR; plasma	20.6%				
				NGS; oral rinse	75%				
				ddPCR; oral rinse	8.3%				
				qPCR; oral rinse	2.1%				
Siravegna et al. (2022) [65]	Comparing effectiveness of a non-invasive diagnostic approach using ctHPVDNA liquid biopsy and imaging/physical exam to a standard diagnostic clinical workup with tumor biopsy	HPV-positive HNSCC (oropharyngeal, nasopharyngeal, and sinonasal SCCs)	131, 61 patients with HPV-positive HNSCC, 45 controls with HPV-negative HNSCC, and 25 healthy controls	ddPCR; plasma	98.4%	98.6%	Standard clinical workup with tumor biopsy	Non-invasive techniques with liquid biopsy were significantly more effective in diagnosing HPV-positive HNSCC compared to the current standard of care with tumor biopsy (Youden index of 0.937 vs. 0.070)	Selection and information biases due to observational study design, lack of details present in referenced outside medical records
Wei et al. (2022) [44]	Testing the effectiveness of EC-ARMS-qPCR assay in the detection of *BRAF^V600E^* mutation in ctDNA from plasma samples of patients with PTC	PTC	74; 54 patients with PTC and 20 patients with benign thyroid nodules	EC-ARMS-qPCR assay; plasma	68.42%	85.71%	EC-ARMS-qPCR assay using FNA samples (concordance of 73.08%)	EC-ARMS-qPCR assay can detect *BRAF^V600E^* ctDNA mutations in plasma samples and is in good concordance with test results of EC-ARMS-qPCR assay performed using FNA tissue samples	Small sample size, case–control design, only 26 patients (22 with PTC, 4 with benign thyroid nodules) underwent FNA for comparison
Almubarak et al. (2020) [42]	Assessing the use of liquid biopsy to detect *BRAF^V600E^* mutations in plasma ctDNA of patients with PTC for monitoring of minimal residual tumor presence	PTC	38	3D digital PCR; plasma	86%	90%	Serum Tg (sensitivity of 78%, sensitivity of 65%)	The 3D digital PCR plasma assay using ctDNA had greater sensitivity and specificity for detecting minimal residual PTC tumors than serum Tg levels. The use of both techniques in conjunction could further increase sensitivity and specificity values	Small sample size
Chera et al. (2019) [66]	Determining performance metrics of ddPCR in diagnosing non-metastatic HPV-positive oropharyngeal SCC and testing for disease control in patients post chemoradiotherapy using blood plasma ctDNA	HPV-positive oropharyngeal SCC	103	ddPCR; plasma	89%	97%	Did not have a method to compare to; patients were eligible if they had their diagnoses confirmed by tumor biopsy	ctHPVDNA is detectable in patients with newly diagnosed HPV-positive oropharyngeal SCC and liquid biopsy assays quantifying plasma ctHPVDNA levels can stratify patients by risk in the post-treatment period	Low power due to low rates of disease persistence, disease recurrence, and patient follow-up
Damerla et al. (2019) [67]	Assess effectiveness of ddPCR using plasma ctHPVDNA in the detection of early-stage HPV-associated SCC	HPV-positive oropharyngeal SCC	132; 97 patients with HPV-positive oropharyngeal SCC, 8 patients with HPV-positive anal SCC, 7 controls with HPV-negative oropharyngeal SCC, and 20 healthy controls without cancer	ddPCR; plasma	95.6% (only accounting for oropharyngeal SCC patients)	100% (only accounting for oropharyngeal SCC patients)	p16 immunohistochemistry assays and HPV DNA or RNA in situ hybridization assays using tumor tissue (sensitivity and specificity values not reported)	ctHPVDNA has high sensitivity and specificity for the detection of intact HPV-positive tumors, even in patients with low tumor burden. This implies clinical utility in screening and treatment response monitoring	Did not genotype all pathological specimens to identify specific HPV subtype, patients with low tumor burden had locoregional disease with potential micrometastatic lesions and thus may not be representative of all patients with early subclinical disease
Chan et al. (2017) [68]	Determining utility of screening for nasopharyngeal carcinoma (NPC) using EBV DNA in the plasma of asymptomatic patients	NPC	20,174	qPCR; plasma	97.1%	98.6%	No screening; diagnosis per standard of care using endoscopy and MRI. Screening cohort had a significantly higher proportion of stage I and II disease (71% vs. 20%) and significantly greater rates of 3-year progression-free survival (97% vs. 70%)	Screening asymptomatic individuals for NPC using EBV DNA plasma levels is associated with earlier diagnosis and better outcomes compared to individuals not undergoing screening	Sampling bias as participants were ethnically Chinese men aged 40 to 62 in Hong Kong, where NPC is endemic
Ahn et al. (2014) [69]	Determining effectiveness of liquid biopsy assays using ctHPVDNA from plasma and oral rinses in detecting oropharyngeal SCC prior to beginning treatment	Oropharyngeal SCC	93; 81 patients with HPV-16 positive SCC and 12 with HPV-16 negative SCC	qPCR; plasma and oral rinses	76.1% (combined plasma and oral rinse sample results)	100% (combined plasma and oral rinse sample results)	Compared use of oral rinse samples and plasma samples to one another in effectiveness of corroborating HPV status of tumor biopsy	Using combination of findings from assays analyzing plasma and oral rinse samples increases the sensitivity of HPV-16 liquid biopsy assays in the screening for HPV-positive oropharyngeal SCC compared to use of either sample type alone. Liquid biopsy assays using these samples provide valuable prognostic information on recurrence free survival and overall survival	Small sample size, retrospective design
	Determining effectiveness of liquid biopsy assays using ctHPVDNA from plasma and oral rinses in predicting 3-year recurrence of oropharyngeal SCC				90.7% (combined plasma and oral rinse sample results)	69.5% (combined plasma and oral rinse sample results)			

**Table 4:** Clinical trials examining liquid biopsy use in HNC *.

ClinicalTrials.gov Study ID	Aim	Study Design	Type of HNC	Sample Size	Samples Used	Enrollment Status	Year of Study Start Date	Estimated Year of Study Completion
NCT05969262 [132]	Development of early intervention, detection, and treatment strategies for HNCs using combined proteomic and liquid biopsy techniques for analysis of patient plasma and urine samples	Mixed methods study utilizing a retrospective cohort and prospective cohort	HNCs; histopathological and anatomical subtypes not specified	500; 250 in the retrospective cohort (125 HNC patients, 125 healthy controls) and 250 in the prospective cohort (125 HNC patients, 125 healthy controls)	Plasma and urine	Recruiting	2023	2025
NCT05645783 [133]	Determining the sensitivity and specificity of a NGS liquid biopsy assay in detecting HNSCC in high-risk patients with head and neck lesions	Prospective observational study	HNSCC	170	Blood	Recruiting	2023	2024
NCT05774561 [134]	Evaluate use of liquid biopsy in risk stratification of patients with HPV-positive HNC and cervical cancer according to disease recurrence	Mixed methods study utilizing retrospective and prospective designs; prospective portion includes newly diagnosed patients and retrospective portion includes patients post-treatment follow-up	HPV-positive oropharyngeal carcinoma	480; 200 patients with oropharyngeal cancer and 280 patients with cervical cancer or high-grade cervical intraepithelial lesions	Oropharyngeal swabs, oral rinses, exhaled breath condensate, and blood	Recruiting	2022	2026
NCT05682703 [135]	Examining changes in plasma and urine metabolites of patients with NPC at different points in the disease and treatment course	Observational cohort study	NPC	2000	Plasma and urine	Recruiting	2022	2025
NCT04742608 [136]	Determining the sensitivity and specificity of extracellular vesicles liquid biopsy techniques in the diagnosis of thyroid cancer	Prospective cohort study	Thyroid cancer; histopathological subtype not specified	250	Blood	Suspended	2020	2026
NCT04599309 [137]	Comparing ctDNA and/or ctHPVDNA levels in blood samples of patients with locally advanced HNSCC before and after undergoing standard treatment	Prospective cohort study	Locally advanced stage III/IV HNSCC	35	Blood	Active, not recruiting	2020	2024
NCT04606940 [138]	Characterizing the levels of ctDNA in blood samples of patients with recurrent or metastatic HNSCC following the first dose of anti-PD1 antibody immune checkpoint inhibitor therapy	Prospective cohort study	HNSCC	18	Blood	Completed	2020	2021
NCT04490564 [139]	Establishing performance metrics for liquid biopsy assay that detects PD-L1 expression by CTCs in peripheral blood samples of patients with metastatic/recurrent HNSCC, Non-Small Cell Lung Cancer, or metastatic melanoma	Prospective cohort study	HNSCC	155; 25 patients with metastatic/recurrent HNSCC, 120 patients with Non-Small Cell Lung Cancer, 10 patients with metastatic melanoma, and 30 healthy controls	Plasma	Active, not recruiting	2019	2023
NCT03926468 [140]	Determining diagnostic performance of ddPCR assay measuring ctDNA in peripheral blood samples of patients with stage III/IV HNSCC at baseline and 3 months after completing treatment	Prospective cohort study	Stage III/IV HNSCC	30	Blood	Unknown status	2019	2022
NCT03712566 [141]	Serially characterizing the changes in genomic, epigenetic, and immune profiling attributes of peripheral blood samples obtained from patients with recurrent or metastatic SCC of the head and neck, esophagus, or anus who are undergoing treatment with platinum-based chemotherapy or immunotherapy	Prospective cohort study	Recurrent or metastatic HNSCC	39	Blood	Active, not recruiting	2018	2024
NCT03942380 [142]	Testing if liquid biopsy assays using ctDNA, ctHPVDNA, or ctRNA in blood samples of patients can detect newly diagnosed or recurrent HNSCC	Interventional, non-randomized study	HNSCC	500	Blood	Recruiting	2017	2025
NCT05122507 [143]	Evaluating the use of NGS, ELISA, and PCR assays in monitoring tumor-associated nucleic acids and protein biomarkers to assess patient response to treatment, early detection of recurrence, and overall prognosis	Prospective cohort study	HNSCC	200	Plasma, serum, and saliva	Recruiting	2017	2023

* Results were not made available.
